# Pelvic reconstruction with different rod-screw systems following Enneking type I/I + IV resection: a clinical study

**DOI:** 10.18632/oncotarget.17164

**Published:** 2017-04-17

**Authors:** Peng Lin, Youyou Shao, Huigen Lu, Zhengliang Zhang, Haiqing Lin, Shengdong Wang, Binghao Li, Hengyuan Li, Zhan Wang, Nong Lin, Zhaoming Ye

**Affiliations:** ^1^ Department of Orthopedics, The Second Affiliated Hospital of Zhejiang University School of Medicine/Orthopedics Research Institute of Zhejiang University, Hangzhou 310009, China; ^2^ Department of Orthopedics, Taizhou Hospital of Zhejiang Province, Linhai 317000, China; ^3^ Department of Pediatric Pulmonology, The Second Affiliated Hospital and Yuying Children's Hospital of Wenzhou Medical University, Wenzhou 325027, China; ^4^ Department of Orthopedics, The Second Hospital of Jiaxing, Jiaxing 314000, China; ^5^ Department of Orthopedics, Dongyang People's Hospital, Jinhua 322100, China

**Keywords:** Enneking type I/I + IV, limb salvage surgery, mechanical failure, pelvic girdle reconstruction, pelvic tumor

## Abstract

The mechanical outcomes of patients with pelvic bone tumors involving zone I or zone I + IV who received resection and different reconstructions are not clear. Therefore, the purpose of this study was to compare the outcomes of different rod-screw systems in reconstruction for these patients, and evaluate the relative risk of mechanical failure for them. We reviewed 30 patients for a mean duration of 40.4 months of follow-up (range, 13.1–162.2 months), five patients had mechanical complications. The mechanical survival rate of two-rod and four-screw (TRFS) group was significantly higher than one-rod and two-screw (ORTS) group (*p* = 0.000). The implant survival rate was correlated with ages (*p* = 0.010), younger people are more likely to fail. Thus, TRFS fixation for pelvic reconstruction after Enneking type I/I + IV resection can provide better short to long-term mechanical stability compared with ORTS fixation, the strength of ORTS fixation is not enough. In addition, biological reconstruction such as autologous bone graft is recommended for the patients who are younger or suffered from benign tumor. As for the patients who are older, with malignant tumors, underwent adjuvant radiotherapy or chemotherapy, functional reconstruction with bone cement is a good choice.

## INTRODUCTION

Reconstruction after resection of pelvic bone tumors involving zone I or zone I + IV remains one of the most demanding procedures. A number of authors recommend limb salvage which can provides better quality of life compared with hemipelvectomy, in spite of the risk of local recurrence [1–3]. Limb-salvage surgeries for pelvic tumors were challenging procedures [4], but a great deal of clinical experience, along with the developments of imaging, adjuvant therapies, surgical techniques and reconstruction materials, have proved such kind of surgeries is feasible for selected patients [5–8]. The methods of the reconstruction of the pelvic girdle are not unified, including simple excision without reconstruction [4, 9], different rod and screw systems [10–13], bone grafts [14–16], bone cement with the plates or cortical bone screws [17, 18], prosthesis [19], etc. The challenge in reconstruction is to providing a solid reconstitute of pelvic girdle and reduce collapse or rotation of the residual portion of the hemipelvis after weight-bearing and remain good function not only in short term but also in long term follow up. Among multifarious reconstruction methods after internal hemipelvectomy (type I or type I + IV), stable internal fixation and pelvic girdle reconstruction allowed early ambulation and provide better short-term to long-term outcomes and function, although the post-operative complications are common [4–7, 20].

Nevertheless, no previous clinical studies have focused on the difference between reconstruction of the pelvic girdle with different rod-screw systems. The purpose of this retrospective study was to review our experience with thirty consecutive cases that underwent reconstructions after Enneking type I/I + IV resection. And to describe the reconstruction with different rod-screw systems, report on the complications and outcomes, evaluate the relative risk of mechanical failure.

## RESULTS

### Patients' demographics, post-operative complications

The mean duration of follow-up was 40.4 months (range, 13.1–162.2 months) at the time of the latest follow-up. The complication rate was about 40% with 12 patients affected. Three had wound complications, one had superficial infection, none of which required further surgery. Four had neurological defects because of the resection of some sacral nerve roots during type IV resection, only one patient (case 4) had almost compensated two years post-operatively. One was found bone nonunion 65.8 months post-operative, with no mechanical failure.

### Oncological results

Eight patients with benign tumors had no local recurrence and distant metastasis. When considering only malignant tumors, the overall metastasis rate was 40.9% (9/22), the local recurrence rate was 45.5% (10/22).

### Mechanical outcomes

**Figure 1:**
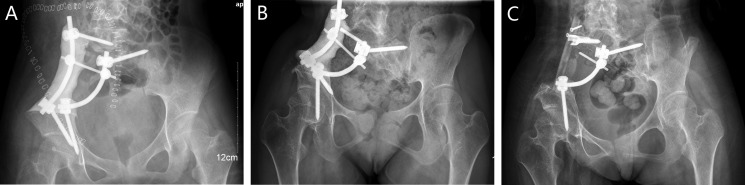
A 16-year-old female (case 4) with diagnosis of pelvis osteosarcoma affecting zone I + IV (**A**) AP radiograph at one week post-operatively. (**B**) AP radiograph at 61.9 months post-operatively, showing failure of the implant, and the implant was stabbed to the skin when she stooped, (**C**) AP radiograph after the construction was revised and augmented with an autologous fibular graft 80 months post-operatively.

**Figure 2:**
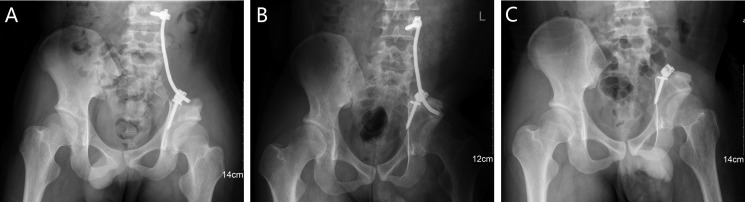
A 20-year-old male (case 25) with diagnosis of pelvis osteosarcoma affecting zone I (**A**) AP radiograph at two weeks post-operatively. (**B**) AP radiograph at 30.5 months post-operatively, showing failure of the implant and with functional impairment. (**C**) AP radiograph at one week after removal of the implant.

**Figure 3:**
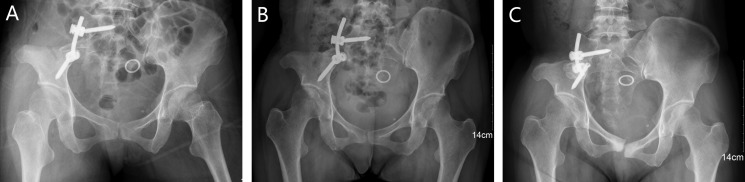
A 37-year-old female (case 26) with diagnosis of pelvis chondrosarcoma affecting zone I (**A**) AP radiograph of the pelvis after bone tumor resection and reconstruction with an autograft bone and screw-rod system. (**B**) AP radiograph showing the implant loosening 3 months post-operatively without functional impairment. (**C**) at 51.3 months post-operatively, AP radiograph showing failure of the implant, pseudoarthrosis formation was found in the resection site, and degeneration was developed in the symphysis pubis.

**Figure 4:**
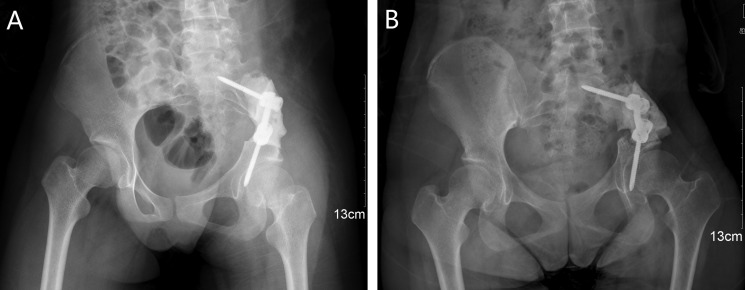
A 15-year-old female (case 28) with diagnosis of pelvis osteosarcoma affecting zone I (**A**) AP radiograph of the pelvis after bone tumor resection and reconstruction with screw-rod system and bone cement. (**B**) AP radiograph showing the implant loosening 14 months post-operatively without functional impairment.

Instrumental survival status were as follows: five patients had mechanical failures and only two patients (case 4, 25) had a revision surgery. Case 4 had a S1 screw breakage (Figure 1B), and the implant was stabbed to the skin when she stooped, in order to improve the stability, the construction was revised and augmented with an autologous fibular graft (Figure 1C). Case 25 had malposition of a loosening rod (Figure 2B), he had a revision surgery to remove the implant (Figure 2C). Case 26 had implant loosening 3 months post-operatively (Figure 3B), and had implant breakage 51.3 months post-operatively (Figure 3C). There was a pseudoarthrosis formation in the resection site and degeneration development in the symphysis pubis at last follow up, she complained of pain in the resection site and symphysis pubis when walking. Case 22 found implant loosening 20.7 months post-operatively, he walked with crutches from then on, and had no further implant breakage. Case 28 found implant loosening 14 months post-operatively (Figure 4B), he stayed in bed for one month and walked with crutches for 3 months, and had no further implant loosening or breakage. The last three patients have varying degrees of dysfunction, but still can be tolerated, so no further surgery was performed.

**Figure 5:**
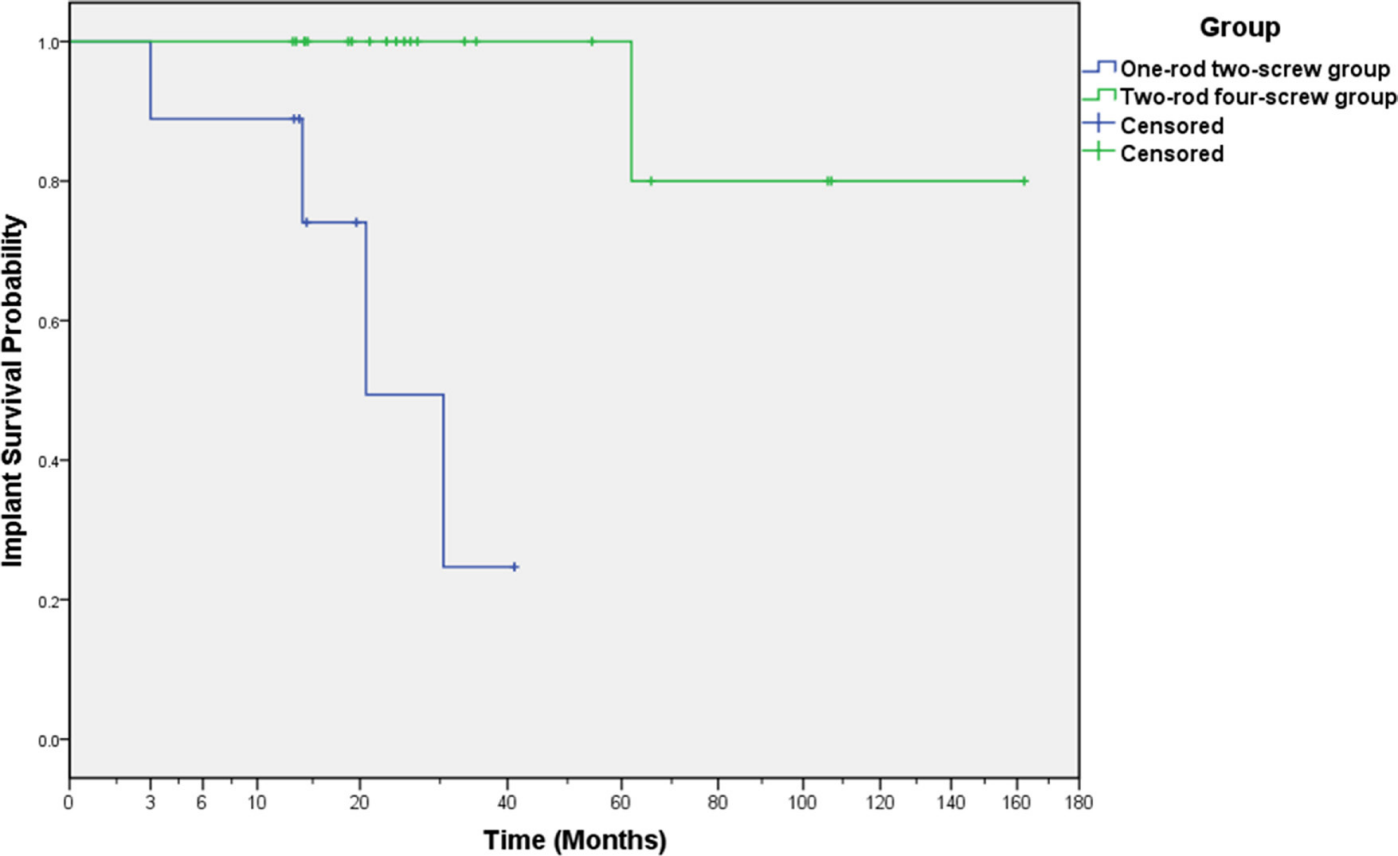
Overall implant survival analysis through Kaplan-Meier for different reconstruction method Number of patients: 30 patients. A statistically significant difference was noted between the one-rod two-screw group and the two-rod four-screw group (*p* = 0.000).

Fourteen patients were alive with no mechanical failure, 11 were died without mechanical failure. The cumulative probability of mechanical failure was 11.1% (95% confidence interval, 1.6% to 56.7%), 11.1% (95% confidence interval, 1.6% to 56.7%), 25.9% (95% confidence interval, 7% to 71.1%), 50.6% (95% confidence interval, 17.7% to 92.2%), and 75.3% (95% confidence interval, 34.8% to 99.0%) at six, twelve, eighteen, twenty-four and thirty-six months, respectively in ORTS group. The cumulative probability of mechanical failure was 0% (95% confidence interval, 0% to 0%) at one to five years, and 20% (95% confidence interval, 3.1% to 79.6%) at six to thirteen years, respectively in TRFS group. The implant survival rate was statistically influenced by the reconstruction with different rod-screw systems. The survival rate of TRFS group was higher than ORTS group (*p* = 0.000), the cumulative implant survival cures of the two groups is shown in Figure 5.

We then compare the overall implant survival rate between the five sub groups (described above), there was statistically difference between each group by the pooled over strata test (*p* = 0.008). We compared the difference between each two groups by pairwise for each stratum test, and found statistically difference between extrapelvic and intrapelvic+extrapelvic group (*p* = 0.001), intrapelvic and intrapelvic+extrapelvic group (*p* = 0.020), extrapelvic and intrapelvic+ intrapelvic group (*p* = 0.034). There was no statistically difference between extrapelvic and intrapelvic group (*p* = 0.910).

The implant survival rate of zone I + IV group was lower than zone I group (*p* = 0.022), there was no statistical difference if exclude the influence of different rod-screw group factor (*p* = 0.447). There was no overall statistically difference between the use of bonegraft, bone cement and none (*p* = 0.054), but the survival rate with bone graft is higher than none (*p* = 0.033). The implant survival rate was correlated with ages by linear trend test (*p* = 0.010), younger people are more likely to fail. The implant survival rate in group of ages less than 22 was lower than group of ages larger than 22 (*p* = 0.000). It was not statistically influenced by chemotherapy (*p* = 0.085), radiotherapy (*p* = 0.150), gender (*p* = 0.730) or BMI (*p* = 0.317).

### Functional outcomes

The mean Musculoskeletal Tumor Society [21] (MSTS) score (%) post-operatively was 81.0% (range, 43 to 97%). The mean MSTS score (%) of the mechanical failure patients post-operatively was 59.4% (SD: 13.5%), the mean MSTS score (%) of the patients with no mechanical failure post-operatively was 85.3% (SD: 9.6%). The MSTS score of the patients with no mechanical failure was significantly higher than mechanical failure patients (*p* = 0.000). The mean MSTS score (%) of the ORTS patients post-operatively was 73.1% (SD: 18.5%), the mean MSTS score (%) of the TRFS patients post-operatively was 84.4% (SD: 10.5%), there was no significantly difference between these two groups (*p* = 0.116).

### Risk factors of post-operative mechanical failures

To investigate the risk factors of post-operative mechanical failures, we firstly did a univariate correlation analysis including 10 factors listed in Table 1 and Table 2. We found that reconstruction with ORTS system was a risk factor (Table 3), as the relative risk was 16 (95% confidence interval, 1.45 to 176.45). Age less than 22 was a risk factor (Table 3), as the relative risk was 96 (95% confidence interval, 4.94 to 1865.70). Zone IV involvement may a potential risk factor (*p* = 0.022) though there was no statistical difference if exclude the influence of different rod-screw group factor (*p* = 0.447), the relative risk was 11 (95% confidence interval, 1.27 to 95.18).

**Table 1:** Patient demographics data and adjuvant therapy details

No.	Age	Gender	Height (cm)	Weight (kg)	BMI	Histologic diagnosis	Bone graft	Bone cement	Chemotherapy	Radiotherapy
**1**	26	Female	158	40.5	16.2	Osteoclastoma	None	Yes	None	None
**2**	35	Male	174	70	23.1	Osteoclastoma	Yes	None	None	None
**3**	48	Female	158	53	21.2	Non-ossifying fibroma	Yes	None	None	None
**4**	16	Female	150	34	15.1	Osteosarcoma	None	Yes	Yes	None
**5**	41	Male	175	65	21.2	Chondrosarcoma	Yes	None	None	None
**6**	44	Female	162	49	18.7	Osteoclastoma	Yes	None	None	None
**7**	39	Male	168	60	21.3	Chondrosarcoma	Yes	None	None	None
**8**	63	Female	149	52	23.4	Chondrosarcoma	Yes	None	None	None
**9**	33	Male	173	65	21.7	Chondrosarcoma	None	Yes	None	None
**10**	59	Male	174	53	17.5	Osteosarcoma	None	Yes	Yes	None
**11**	38	Male	165	55	20.2	Benign	Yes	None	None	None
**12**	68	Male	177	63	20.1	Chondrosarcoma	None	Yes	None	None
**13**	43	Female	161	61	23.5	Osteosarcoma	Yes	None	Yes	None
**14**	48	Female	164	47	17.5	Metastatic Thyroid cancer	None	Yes	None	Yes
**15**	66	Male	172	58	19.6	Metastatic bladder cancer	None	Yes	None	None
**16**	36	Female	164	55	20.4	Osteoclastoma	None	Yes	None	None
**17**	69	Female	167	55	19.7	Metastatic cancer	None	Yes	Yes	None
**18**	15	Male	165	35	12.9	Chondrosarcoma	Yes	None	Yes	None
**19**	59	Female	164	48	17.8	Osteosarcoma	None	None	Yes	None
**20**	28	Male	174	52	17.2	Chondrosarcoma	Yes	None	None	None
**21**	44	Female	165	55	20.2	Malignant fibrous histiocytoma	None	None	Yes	None
**22**	19	Male	172	60	20.3	Fibrosarcoma	None	None	None	None
**23**	34	Male	172	55	18.6	Solitary fibrous tumor	Yes	None	None	None
**24**	24	Male	173	56	18.7	Ewing's sarcoma	Yes	None	Yes	Yes
**25**	20	Male	172	64	21.6	Osteosarcoma	None	None	Yes	None
**26**	37	Female	165	60	22.0	Chondrosarcoma	Yes	None	None	None
**27**	32	Female	161	50	19.3	Chondrosarcoma	Yes	None	None	None
**28**	15	Female	159	50	19.8	Osteosarcoma	None	Yes	Yes	Yes
**29**	61	Male	165	55	20.2	Aneurysmal bone cyst	None	Yes	None	None
**30**	62	Male	174	58	19.2	Metastatic malignant melanoma	None	None	Yes	None

**Table 2:** Surgical details, complications and the outcomes of the patients

No.	Type of resection	Site of fixation (proximal/distal)	Method of fixation	Type of fixation	MSTS score (%)	Complications	mechanical survival time (months)	Mechanical outcome	Follow-up period (months)
1	I	Vertebral pedicle (L5), sacrum/ periacetabular bone	2 rods 4 screws	EXP + INP	97		162.2	AMF	162.2
2	I	Transverse process (L5), sacrum/ periacetabular bone	2 rods 4 screws	EXP + INP	90		107.1	AMF	107.1
3	I	Transverse process (L5, sacrum)/periacetabular bone	2 rods 4 screws	EXP + INP	90		106.2	AMF	106.2
4	I + IV	Transverse process (L5), sacrum/ periacetabular bone	2 rods 4 screws	EXP + INP	77	Neurological defects, metal breakage	61.9	MF	84.5
5	I	Transverse process (L5), sacrum/periacetabular bone	2 rods 4 screws	EXP + INP	87	Bone nonunion	65.8	DMF	65.8
6	I	Transverse process sacrum/ periacetabular bone	2 rods 4 screws	INP + INP	97		54.4	AMF	54.4
7	I	Transverse process sacrum/ periacetabular bone	2 rods 4 screws	INP + INP	87	Superficial infection	35.2	DMF	35.2
8	I	Vertebral pedicle (L5), sacrum/ periacetabular bone	2 rods 4 screws	INP+EXP	87		33.5	AMF	33.5
9	I	Transverse process (L5), sacrum/ periacetabular bone	2 rods 4 screws	INP+EXP	83		27.0	DMF	27.0
10	I	Transverse process (L5), sacrum/ periacetabular bone	2 rods 4 screws	EXP + INP	83	Wound complication	26.1	DMF	26.1
11	I	Transverse process sacrum/ periacetabular bone	2 rods 4 screws	INP + INP	93.3		25.3	AMF	25.3
12	I	Transverse process sacrum/ periacetabular bone	2 rods 4 screws	INP + INP	87		24.3	DMF	24.3
13	I	Transverse process (L5), sacrum/ periacetabular bone	2 rods 4 screws	EXP + INP	90		23.1	DMF	23.1
14	I	Transverse process sacrum/ periacetabular bone	2 rods 4 screws	INP + INP	63.3		21.1	DMF	21.1
15	I	Transverse process sacrum/periacetabular bone	2 rods 4 screws	INP + INP	80	Wound complication	14.5	AMF	14.5
16	I	Transverse process (L5),sacrum/periacetabular bone	2 rods 4 screws	EXP + INP	90		14.2	AMF	14.2
17	I	Transverse process sacrum/ periacetabular bone	2 rods 4 screws	INP + INP	77		14.2	DMF	14.2
18	I	Vertebral pedicle (L5), sacrum/ periacetabular bone	2 rods 4 screws	EXP + INP	97		13.1	AMF	13.1
19	I + IV	Vertebral pedicle (L5, S1)/ periacetabular bone	1 rod 4 screws	EXP + EXP	67	Neurological defects, deep venous thrombosis	19.1	AMF	19.1
20	I	Vertebral pedicle (L5, S1)/ periacetabular bone	1 rod 4 screws	EXP + EXP	90		18.7	DMF	18.7
21	I + IV	Vertebral pedicle (L4, 5) / periacetabular bone	1 rod 4 screws	EXP + EXP	60	Neurological defects	13.4	DMF	13.4
22	I + IV	Vertebral pedicle (L4, 5)/ periacetabular bone	1 rod 3 screws	EXP	60	Neurological defects, implant loosening	20.7	MF	41.0
23	I + IV	Transverse process (L5)/ periacetabular bone	1 rod 3 screws	EXP	83		19.6	AMF	19.6
24	I	Vertebral pedicle (L5, S1)/ periacetabular bone	1 rod 3 screws	INP	87		14.4	AMF	14.4
25	I + IV	vertebral pedicle (L4)/periacetabular bone	1 rod 2 screws	EXP	43	Implant loosening	30.5	MF	67.3
26	I	Transverse process sacrum/ periacetabular bone	1 rod 2 screws	INP	50	Metal breakage	3.0	MF	67.2
27	I	Transverse process sacrum/ periacetabular bone	1 rod 2 screws	INP	90		41.1	AMF	41.1
28	I	Transverse process sacrum/ periacetabular bone	1 rod 2 screws	INP	67	Implant loosening	14.0	MF	32.1
29	I	Transverse process sacrum/periacetabular bone	1 rod 2 screws	INP	91		13.7	AMF	13.7
30	I	Vertebral pedicle (L5)/ periacetabular bone	1 rod 2 screws	EXP	87	Wound complication	13.2	DMF	13.2

INP: intrapelvic, sacral-pelvic reconstruction, EXP: extrapelvic, lumbo-pelvic reconstruction, MSTS: Musculoskeletal Tumor Society, AMF: alive with no mechanical failure, DMF: died with no mechanical failure, MF: mechanical failure.

**Table 3:** Potential risk factors of post-operative implant failures in univariate analysis

Variables	Mechanical failure No. (%)	Non-mechanical failure No. (%)	*p* value
Rod-screw system			0.000*
One-rod two-screw (ORTS)	4 (44.4)	5 (55.6)	0.008^*^
EXP	2 (50)	2 (50)	0.910^#^
INP	2 (40)	3 (60)	
Two-rod four-screw (TRFS)	1 (4.8)	20 (95.2)	
EXP + EXP	0 (0)	3 (100)	
INP + EXP	1 (9.1)	10 (90.9)	0.001^##^
INP + INP	0 (0)	7 (100)	0.034^##^
Tumor location			0.022
I	2 (8.3)	22 (91.7)	
I + IV	3 (50)	3 (50)	
Reconstruction			0.054
Bone graft	1 (7.1)	13 (92.9)	0.033^###^
Bone cement	2 (18.2)	9 (81.8)	0.122^###^
None	2 (40)	3 (60)	
Chemotherapy			0.085
Yes	3 (27.3)	8 (72.7)	
None	2 (10.5)	17 (89.5)	
Radiotherapy			0.150
Yes	1 (33.3)	2 (66.7)	
None	4 (14.8)	23 (85.2)	
Gender			0.730
Male	2 (12.5)	14 (87.5)	
Female	3 (21.4)	11 (78.6)	
Age (years)			0.000
< 22	4 (80)	1 (20)	
> 22	1 (4)	24 (96)	
BMI			0.317
< 19.75	1 (7.1)	13 (92.9)	
> 19.75	4 (25)	12 (75)	
All	5 (16.7)	25 (83.3)	

Significant at a = 0.05 *significant difference between ORTS and TRFS group, ^*^significant difference between five sub groups by the pooled over strata test, ^#^compared with INP group by pairwise for each stratum test, ^##^compared with EXP group by pairwise for each stratum test, ^###^compared with None group by pairwise for each stratum test. (Kaplan-Meier survival analysis, Log rank test was used to compare implant survival rates between different groups). INP: intrapelvic, sacral-pelvic reconstruction, EXP: extrapelvic, lumbo-pelvic reconstruction.

## DISCUSSION

The traditional hemipelvectomy in treating pelvic tumors was replaced by limb-salvage surgery, which was combined with adjuvant therapy since the similar survival and recurrence rates [22–24]. Limb-salvage surgery can improve quality of life, reduce psychological trauma and physical disability, which becomes a favorable procedure. With the development of surgical technique and implant, although the simple resection without reconstruction has certain effect, it is gradually abandoned. Reconstruction with the screw and rod system is the most common choice on account of its reliability and effectiveness. However, mechanical failure was a common complication following internal hemipelvectomy, no matter which reconstruction method has been chosen [9, 10, 12, 25]. To date, there are only two articles using finite element analysis of the biomechanics to evaluate different methods of rod-screw systems [26, 27]. In our cohort, we compared the effects of different rod-screw fixations on the prognosis, and found inadequate fixation of the pelvic girdle is likely the main reason in the mechanical failures.

**Figure 6:**
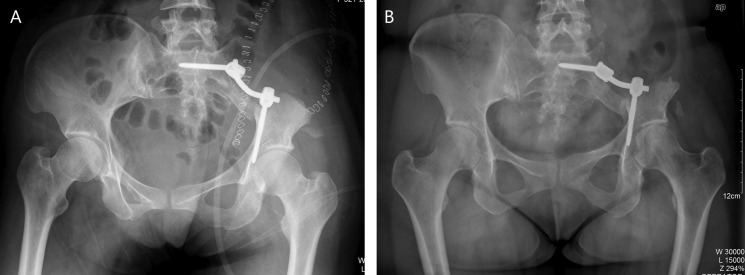
A 32-year-old woman (case 27) with diagnosis of pelvis chondrosarcoma affecting zone I (**A**) AP radiograph of the pelvis after internal hemipelvectomy and subsequent pelvic ring reconstruction with screw-rod system and autograft bone. (**B**) AP radiograph showing the bone union 12.3 months post-operatively.

ORTS fixation has been used by many authors, and achieved good outcomes [10, 11]. In our series, we adopt the similar method as it reports [10], although there were several satisfactory results (Figure 6B), the total abortion incidence was significantly higher than TRFS fixation, we considered this reconstruction method is insufficient as the torsional stability is not enough (Figures 2B, 3B–3C, 4B). One literature introduced TRFS fixation, they believe the mechanical strength of TRFS system is good, of course, if coupled with bone cement or bone graft, the initial stability will be better, this conclusion is similar to our result [13]. We performed the TRFS fixation as previously reported [12], there is only one patients (4.8%) suffered implant breakage 61.9 months post-operatively when she was 16 years old at the time of the index reconstruction with the bone tumor involving zone I + IV(Figure 1B). We found the implant survival rate was correlated with ages in our cohort (*p* = 0.010), younger people are more likely to fail, because the younger patients with longer survival period, they exercise more, need higher life quality and require better implant intensity. For these younger patients and the primary tumor which has a good life expectancy, biological reconstruction with bone graft can improve their long-term stability.

**Figure 7:**
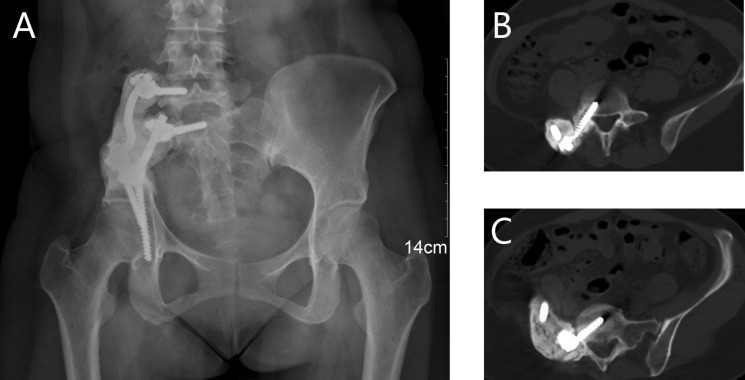
A 26-year-old female (case 1) with diagnosis of pelvis osteoclastoma affecting zone I (**A**) AP radiograph of the pelvis shows internal hemipelvectomy and subsequent pelvic ring reconstruction with screw-rod system and bone cement. (**B**, **C**) CT images show no evidence of tumor recurrence or implant loosening 144.1 months post-operatively.

Intrapelvic fixation is more stable than extrapelvic fixation. The reconstruction of extrapelvic fixation is easy to cause the failure in the long-term follow-up, because of the existence of micromotion between intervertebral discs (i.e., L4/5, L5/S1), the internal fixation stress will increase [27] and the internal fixation will be easy to get fatigue broken or loosening. Case 4 is a good example, there's another case (case 25) in our series, who had the lumbar 4 to iliac bone fixation, the implants loosened 30.5 months post-operatively. We would recommend to achieve intrapelvic fixation if possible mainly for the mechanical stability. But in some cases, it's difficult to put two screws into the sacrum, because the space for the screws in sacrum is limited. Therefore, only one screw could fixed in the sacrum, and the other one is fixed on the lumbar 5 vertebra, as we described as extrapelvic+intrapelvic fixation (Figures 1A, 7A–7C). Zone IV involvement may a potential risk factors (*p* = 0.022), partly because of the extrapelvic fixation due to no enough position for the screws in sacrum. Compared with pedicle screws, lateral lumbar/sacrum vertebral body screws are recommended, as this front column support has biomechanical advantages [28].

**Figure 8:**
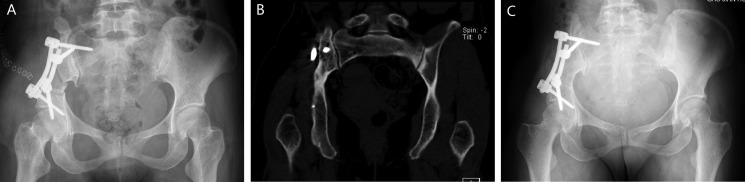
A 44-year-old woman (case 6) with diagnosis of pelvis osteoclastoma affecting zone I (**A**) AP radiograph of the pelvis after bone tumor resection and reconstruction with an autograft bone in the pelvis stabilized with the implantation of screw-rod system. (**B**) Coronal CT image showing the bone union 17.5 months post-operatively. (**C**) AP radiograph of the pelvis shows no evidence of tumor recurrence or implant loosening 36 months post-operatively.

Most of the literatures recommend biological reconstruction, although there is nonunion, bone graft fracture [29], infection [30–32], etc., since its long-term benefits [10, 11, 14, 15, 22, 25, 33–35]. When doing the biological reconstruction after resection of pelvic bone tumors involving zone I or zone I + IV, the iliac crest bone graft on the same side of the host is the first choice (gluteus medius muscle pedicle iliac bone graft [10], vascularized [15] or non-vascularized [11] iliac bone graft) (Figure 8A). When compared with the autogenous fibula graft, it has the advantages of convenient to harvest and minimal invasive. Whereas reconstructions with iliac crest have limitations, as the remainder of the iliac bone is limited when requiring extensive bone resection. Bone grafts such as non-vascularized or vascularized fibular grafts are commonly used to reconstruct large bone defects after resection of the tumor (Figure 1C) [14, 25, 33–35]. There is a high rate of infection and nonunion reported in the allograft fibular transplantation [30, 32], so we do not recommend this technique as first choice. Excellent outcomes were achieved by reconstruction with vascularized fibular grafts in the literatures [34–36]. Nevertheless, this technique requires more complex procedures, longer operative time, greater surgical trauma, and higher donor site morbidity [37]. Non-vascularized fibular grafts are likely to have lower donor site morbidity and are less complex to implant [25], in spite of reservations a lack of biological activity, risk of resorption [38], and require a slightly longer time to union [39]. As a compromised approach, in our view, non-vascularized fibular grafts are the useful alternative to vascularized grafts, especially in sacral iliac region with adequate blood supply and soft-tissue coverage.

Although biological reconstruction is a mainstream, functional reconstruction with rod-screw system and bone cement is recommended for the patients underwent adjuvant radiotherapy or chemotherapy. Because adjuvant radiotherapy or chemotherapy may cause nonunion after bone graft implant, they may have a negative impact on the stability of the biological reconstruction [11, 14].

Several factors need to be considered when selecting the most appropriate reconstructive procedure. For the younger patients, biological reconstruction has great advantages in long term stability (Figure 6B, 8B), compared with non-biological reconstruction (Figure 1B) [10, 14, 15, 25, 33–35]. For the older patients who can't stay in bed for a long time because of poor general condition, bone cement is recommend for the instantaneous mechanical stability, and the patients can get early mobilization [12].

In terms of the oncological outcomes, it was thought that primary or metastatic malignancies, which have a poor life expectancy, should be differentiated from benign tumors. Patient selection criteria, therefore, should take account of oncological outcomes, patients with pelvic malignancies would be recommended to use bone cement mainly for the instantaneous mechanical stability. While treating with benign tumors like osteoclastoma or the primary malignancies which have a good life expectancy like chondrosarcoma, however, autologous bone graft is recommended because this is a biological reconstruction method which has good long-term mechanical stability (Figures 6, 8).

Our study has some limitations such as the limited number of patients, so we include all patients whose data we have collected no matter the length of follow-up. This was however a retrospective studies on patients, so the confounding factors can't be fully controlled. The time of follow up ranged widely, because of the progress of malignant tumor is rapidly. Accordingly, the long-term mechanical failure rate was underestimate the genuine rates.

In conclusion, TRFS fixation for pelvic reconstruction after Enneking type I/I + IV resection can provide better short to long-term mechanical stability compared with ORTS fixation, the strength of ORTS fixation is not enough. In addition, biological reconstruction such as autologous bone graft is recommended for the patients who are younger or suffered from benign tumor. As for the patients who are older, with malignant tumors, underwent adjuvant radiotherapy or chemotherapy, functional reconstruction with bone cement is a good choice for the excellent instantaneous mechanical stability.

## MATERIALS AND METHODS

### Data collection

We retrospectively reviewed our orthopedic database and identified 41 patients who had underwent internal hemipelvectomy of type I or type I + IV for bone tumors at our institution between 2003 and 2015. Of these 11 patients undergoing pelvic resection and reconstruction with insufficient data were excluded from the analysis.

The clinical data, therapy details, and outcomes of the patients were collected. Radiography, CT and MRI studies were used to evaluate the site of the tumor involved and the mechanical outcomes. The study was approved by the ethics committee of the authors' institution.

The study group included 16 male and 14 female with a mean age of 40.7 years (range, 15 to 69 years), a mean body weight of 54.5 kg (range, 34 to 70 kg), a mean height of 166.4 cm (range, 149 to 177 cm), and a mean BMI of 19.6 (range, 12.9 to 23.5) at the time of the index reconstruction. The demographic data and adjuvant therapy details are show in Table 1.

### Specific illustration

The initial tumor location and type of surgical resection was classified as previously reported [4]. A type I lesion involves resection of the ilium, a type IV lesion refers to the lesions involving a portion of sacrum. Combination of type I and type IV depends on the extent of bony invasion by tumor. Implant failure was defined as the breakage or loosening of the implant.

We include the one rod and three screws patients which had one screw fixed in pelvis and two screws in lumbo/sacral vertebra in ORTS group, as for only one screw fixed in supraacetabular. We then include the one rod four screws patients in TRFS group, because of two screws fixed in supraacetabular and lumbo/sacral vertebra respectively.

We define the multiaxial pedicle screws placed through lateral surface of vertebral bodies or pedicle of L4 or L5 as extrapelvic fixation (Figure 2A), and define the screws placed through lateral surface of vertebral bodies or pedicle of sacrum as intrapelvic fixation (Figures 3, 4, 6A). Finally, we divide the ORTS group into two groups: extrapelvic group and intrapelvic group; divide the TRFS group into three groups: extrapelvic+extrapelvic group, extrapelvic+intrapelvic group (Figure 1A, 7A), intrapelvic+intrapelvic group (Figure 8A) depends on the place of the screws. The surgical details, complications and the outcomes of the patients are show in Table 2.

### Surgical procedure

The resection of the pelvic bone tumor is in accordance with Enneking [40]. Two experienced surgeons performed these surgeries and the procedures. Multiaxial pedicle screws (Click'x, Synthes, Switzerland or M8, Medtronic, USA) were placed through pedicle or lateral surface of vertebral bodies of L4, L5 and sacrum. One or two titanium rods were then used to connect the screws, similar to the methods introduce in the literatures [10, 12]. The lateral lumbar/sacral vertebral body screw or the vertebral pedicle screw connecting to supraacetabular screw were sometimes applied as indicated (Figures 1, 2, 3, 4, 6, 7, 8).

Part of the patients were only reconstructed with rod-screw system (Figure 2A), while some were also encased in antibiotic-impregnated bone cement (Figures 1A, 4A, 7A). The rest were reconstructed with rod-screw system coupled with autologous bone graft (Figures 1C, 3A, 6A, 8A), such as fibular grafts, iliac crest or bone graft in titanium cages. In some cases, we fixed the bone grafts to the ilium or sacrum by cortical bone screws to increase stability (Figure 1C).

A combination of type I and IV surgical resection was performed in 6 cases. Specific resection ranges of type IV in these 6 cases were introduced as follows, case 4 was received right sagittal hemisacrectomy combined with transverse partial sacrectomy, the right sacral roots 1–5 and left sacral roots 4–5 were sacrificed; case 19 was received right sagittal hemisacrectomy, the right sacral roots 1–3 were sacrificed; case 21 was received left sagittal hemisacrectomy combined with transverse partial sacrectomy, the left sacral roots 1–5 and right sacral roots 3–5 were sacrificed; case 22 was received right sagittal hemisacrectomy, the right sacral roots 1–3 were sacrificed; case 23 was received right partial sacral ala resection; case 25 was received left partial sacral ala resection.

### Statistical analysis

Kaplan-Meier survival analysis was performed to estimate overall mechanical survival, in which the event was defined as mechanical failure for any reason. Surviving patients or died patients with no implant failure were censored at the last date of follow-up in the analysis of overall mechanical survival. Log rank test was used to compare implant survival rates between different groups. Pooled over strata test was used to compare the overall difference between each groups, and the pairwise for each stratum test was used to compare the difference between each two groups. Cox regression was used to analysis the correlation ship between the implant survival rate and the ages. A *p*-value of less than 0.05 was considered to be significant. All statistical analyses were performed using the Statistical Package for the Social Science (SPSS) software, version 22.0 (SPSS Inc., Chicago, IL, USA).
